# Different Associations between Auditory Function and Cognition Depending on Type of Auditory Function and Type of Cognition

**DOI:** 10.1097/AUD.0000000000000700

**Published:** 2019-08-23

**Authors:** Henrik Danielsson, Larry E Humes, Jerker Rönnberg

**Affiliations:** 1 Department of Behavioural Sciences and Learning, Linköping University, Linköping, Sweden; 2 Linnaeus Centre HEAD, Swedish Institute for Disability Research, Linköping University, Linköping, Sweden; 3 Department of Speech and Hearing Sciences, Indiana University, Bloomington, Indiana, USA

**Keywords:** Cognitive speed, Episodic long-term memory, Gap detection, Hearing loss, Semantic long-term memory, Temporal order, Working memory

## Abstract

Previous studies strongly suggest that declines in auditory threshold can lead to impaired cognition. The aim of this study was to expand that picture by investigating how the relationships between age, auditory function, and cognitive function vary with the types of auditory and cognitive function considered.

**Design:**

Three auditory constructs (threshold, temporal-order identification, and gap detection) were modeled to have an effect on four cognitive constructs (episodic long-term memory, semantic long-term memory, working memory, and cognitive processing speed) together with age that could have an effect on both cognitive and auditory constructs. The model was evaluated with structural equation modeling of the data from 213 adults ranging in age from 18 to 86 years.

**Results:**

The model provided good a fit to the data. Regarding the auditory measures, temporal-order identification had the strongest effect on the cognitive functions, followed by weaker indirect effects for gap detection and nonsignificant effects for threshold. Regarding the cognitive measures, the association with audition was strongest for semantic long-term memory and working memory but weaker for episodic long-term memory and cognitive speed. Age had a very strong effect on threshold and cognitive speed, a moderate effect on temporal-order identification, episodic long-term memory, and working memory, a weak effect on gap detection, and nonsignificant, close to zero effect on semantic long-term memory.

**Conclusions:**

The result shows that auditory temporal-order function has the strongest effect on cognition, which has implications both for which auditory concepts to include in cognitive hearing science experiments and for practitioners. The fact that the total effect of age was different for different aspects of cognition and partly mediated via auditory concepts is also discussed.

## Abstract 2

Supplemental Digital Content is available in the text.

## INTRODUCTION

There is substantial evidence that as people age, declines in both hearing (ISO 2017; Cruickshanks et al. 2010) and cognition (Rönnlund et al. 2005; Salthouse 2010) will co-occur, based on group data. Many studies have also shown a relationship between hearing loss and cognitive decline when controlling for age (Lin et al. 2011; Rönnberg et al. 2011; Harrison Bush et al. 2015). Across studies, the type of cognition examined has varied, but for hearing loss it is typically only pure-tone hearing thresholds (further on shortened to hearing thresholds) that has been measured. The purpose of the present study was to evaluate how age and auditory function affects cognition when multiple types of auditory and cognitive function are measured. The novel aspect of this study was to use nonthreshold auditory tasks together with a modeling approach. More specifically, three types of auditory function were investigated: threshold; temporal order; and gap detection. In addition, four types of cognitive constructs were measured: episodic long-term memory; semantic long-term memory; working memory; and cognitive processing speed.

Cognitive speed starts to show age-related decline early and declines at a higher rate than memory (Salthouse 2009). Of the memory systems, semantic long-term memory is generally regarded as being more stable across the adult life span, peaking at a higher age than episodic long-term memory, even though there are differences between studies (see Rönnlund et al. 2005 for a review). The degree of age-related decline in short-term memory and working memory is dependent on the type of task (Nilsson 2003) and is higher with higher cognitive demands (Salthouse 1994). It is also suggested that dealing with higher demands could be mediated via cognitive speed (Salthouse 1994).

The empirical evidence supports a causal link from auditory acuity to cognitive performance in older adults (but not vice versa), as concluded in three separate reviews of the literature (Rönnberg et al. 2013; Wayne & Johnsrude 2015; Roberts & Allen 2016). It should, however, be noted that this is based on cross-sectional studies. The association between sensory and cognitive decline has been known for decades (Baltes & Lindenberger 1997; Schneider & Pichora-Fuller 2000; Humes et al. 2013). It has been proposed that there is a common age-related decline in sensory processing and cognitive processing, the so-called common cause hypothesis (Lindenberger & Baltes 1994; Baltes & Lindenberger 1997). This is, however, difficult to evaluate because age-related decline in different types of cognition starts at different ages and has different trajectories or rates of decline (Nyberg et al. 2003; Rönnlund et al. 2005). Another complicating factor is that the relationship between hearing thresholds and cognition is different for different types of cognition (Rönnberg et al. 2011). In the study by Rönnberg et al. (2011), the division of cognition was based on theories of memory systems, including short-term memory, episodic long-term memory, and semantic long-term memory. They found associations between hearing loss and semantic as well as episodic long-term memory but not with short-term memory. This pattern of results is relatively consistent with the findings of other studies (Lin et al. 2011; Deal et al. 2015), although these studies were not framed in the same cognitive terminology. Both of these studies found an association between hearing loss and impaired episodic long-term memory but not between hearing loss and impaired short-term memory or word fluency (often interpreted as an index of semantic long-term memory). Harrison Bush et al. (2015) found a small but significant effect of threshold on 10 out of 11 of the cognitive measures they assessed.

Several hypotheses for understanding the causal direction between sensory acuity and cognitive performance in old age have been proposed (see Wayne & Johnsrude 2015 for a review). There are different hypotheses that suggest that sensory decline causes cognitive decline: the Disuse hypothesis that the Ease of Language Understanding model is based on (Rönnberg et al., 2013), the Information Degradation hypothesis (Schneider & Pichora-Fuller 2000; Pichora-Fuller 2003), and the Sensory Deprivation hypothesis (CHABA 1988). To summarize, the empirical evidence from cross-sectional data support a causal link from auditory acuity to cognitive performance in older adults when other factors like age have been accounted for.

Studies of relationships between cognition and other types of hearing measures are rare even though the need for these types of studies is recognized (Humes et al. 2013; Wayne & Johnsrude 2015; Roberts & Allen 2016). Rönnberg et al. (2016) have shown that auditory temporal fine structure measures were more strongly related to cognition than hearing acuity. The overall picture shows that relationships between hearing thresholds and episodic long-term memory are consistently found, but the relationships between hearing thresholds and other types of cognition are found in some, but not all studies. The causal direction of the relationship that has the strongest empirical support, as shown in the literature review earlier, is that auditory decline leads to cognitive decline. It is also suggested that cognition is more strongly related to other types of auditory measures (e.g., temporal aspects) compared with thresholds, although more studies are needed in this area.

Humes et al. (2013) examined the associations among sensory-processing measures in three senses (hearing, vision, and touch) and cognitive function. A principal components factor analysis of the sensory-processing measures revealed the three sensory domains or abilities to be examined here: threshold, temporal-order identification, and gap detection. These three auditory abilities, moreover, tap three of the four auditory abilities (identification of highly familiar sounds not included here) identified in a large sample (N = 338) of normal-hearing young adults completing 18 different tests of auditory performance (Kidd et al. 2007). Humes et al. (2013) found that these auditory abilities were largely separate from similar measures for vision and touch, based on the majority of cross-factor correlations being weak in the first-order factor analysis. However, there was also a shared variance across all of these measures tying them together, based on the emergence of a single factor, “global sensory processing,” a the second-order factor analysis. This factor was strongly correlated with cognitive function, independent of age. The present study, using a subset of the data from the study by Humes et al. (2013), has the novel aspect of including nonthreshold auditory tasks to model how age, auditory functions, and cognition are related to one another. The present study provides a more detailed examination of the associations between three constituent auditory-processing measures (hearing threshold, auditory temporal-order identification, and auditory gap detection threshold) and four constituent cognitive processing measures (episodic long-term memory, semantic long-term memory, working memory, and cognitive processing speed). Given the focus on only the auditory measures in these analyses, a “global” sensory-processing factor integrating auditory, visual, and tactile measures from the study by Humes et al. (2013) was not examined here. With regard to the four constituent cognitive processing measures used here, these were guided by grouping of the measures into various scales of the Wechsler Adult Intelligence Scale (Wechsler 1995), as well as prior work on episodic memory (Rönnberg et al. 2011, 2013) (Table 3)

### Modeling the Inter-Relationships Among Age, Hearing, and Cognition

The present study uses structural equation modeling (SEM). SEM combines measured variables and latent (unmeasured) variables in the same model and is advantageous compared with many simpler approaches. For example, three different measures of auditory thresholds, at different frequencies, can be included as indicators of the latent variable auditory threshold. Models with specific inter-relationships among variables can be specified, estimated, and evaluated (see Anderson & Gerbing 1988; Bentler 1988; Jöreskog & Sörbom 1993). SEM is widely used in aging research and guidelines for reporting of SEM results were published as early as 1991 (Raykov et al. 1991). SEM requires relatively large datasets (Enmarker et al. 2006) and allows for objective evaluation of the adequacy of fit of a theoretical model to data. Theory testing and causal modeling in SEM are not enough for making causal inference. Rather, assumptions concerning the study context and data are required (see Mulaik 1987; Bentler 1989). On the basis of the literature review earlier, assumptions regarding the direction of causality are as follows: 1. age can cause change in all the (auditory and cognitive) latent variables; and 2. decline in auditory function can cause decline in cognition, but not the other way around.

**Fig. 1.**
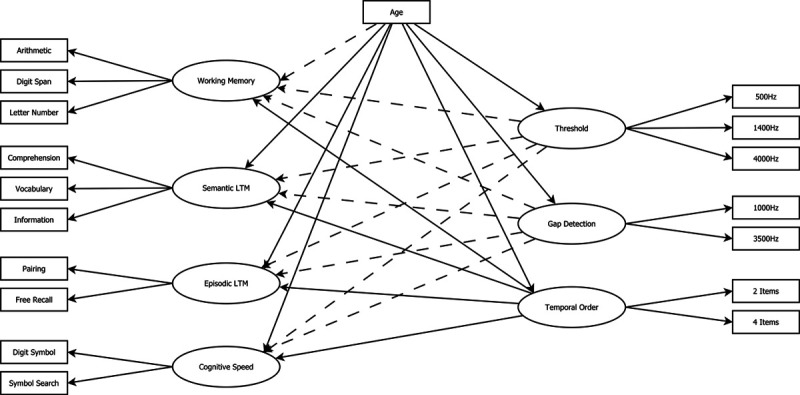
How the modeling of age, the four auditory functions, and the three cognitive functions were set up. All paths in the figure were included in the modeling, but in the final model only the paths with solid line were kept and the paths with the dashed lines were excluded.

In the present study, one structural equation model will examine specific predictions on the inter-relations between age, three different types of auditory function (threshold, temporal-order identification, and gap detection), and four types of cognitive functions (episodic long-term memory, semantic long-term memory, working memory, and cognitive processing speed) (see Figure 1 for an overview of the starting model). On the basis of the literature review, which is typically confined to hearing thresholds as the sole measure of auditory function, we predict hearing thresholds will have effect on episodic long-term memory, and semantic long-term memory but not on working memory or cognitive speed. On the basis of the few studies that have included other auditory measures than hearing thresholds, it was predicted that the effect on cognition auditory should be larger for temporal-order function compared with auditory threshold. As deficits in auditory temporal-order identification and gap detection simply represent additional forms of degradation of the auditory input, akin to hearing thresholds, it can also be predicted that the effects on cognition should be of the same magnitude for all the auditory measures. This study, however, will be able to determine the validity of this assumption and the resulting predictions.

## MATERIALS AND METHODS

### Participants

A total of 245 young, middle-aged and older adults participated in the study by Humes et al. (2013). The present data of 213 participants represent a subset of the original data for whom no data were missing for any of the measures of interest for this study. The first age group consisted of 49 young adults (39 females and 10 males) with a mean age of 22.7 years (range = 18 to 30 years), the second group consisted of 38 middle-aged adults (25 females and 13 males) with a mean age of 48.3 years (range = 40 to 55 years), and the third group consisted of 126 older adults (72 females and 54 males) with a mean age of 70.7 years (range = 60 to 87 years). Participants were recruited for this study via advertisements in the local newspaper, in bulletins and flyers for local community centers and organizations, and posted in various locations on the campus of Indiana University, Bloomington, Indiana. Selection criteria were based on age (18 to 35 years for the young adults, 40 to 55 years for the middle-aged adults, and 60 to 89 years for the older adults), a score ≥ 25 on the Mini-Mental State Examination (Folstein et al. 1975), and several sensory measures. These criteria are briefly described later; see Humes et al. (2013) for more details. Maximum acceptable hearing thresholds and allowable visual acuity were established and used in subject selection. These limits were not designed to be particularly selective but to ensure that the stimuli would most likely be visible and audible when presented on subsequent tasks. This was confirmed directly via identification screening. All participants were required to pass an identification screening of the four auditory vowel stimuli in isolation, used in subsequent temporal-order measurements, with at least 90% accuracy on one of up to four, 20-trial blocks.

As expected, several older subjects and some middle-aged subjects had measurable hearing loss in the higher frequencies. The mean high-frequency (1000, 2000, and 4000 Hz) HLs, averaged across both ears, were 7.0, 16.4, and 26.6 dB HL for the young, middle-aged, and older groups, respectively. There were no significant differences in average hearing loss for either ear in any given group. The default ear for auditory testing was the right ear, but six older adults had a slight asymmetry in hearing thresholds such that the left ear met our selection criteria for inclusion in the study, but the right ear did not. Thus, for this age group, 6 subjects had their left ear tested and 120 had their right ear tested on the auditory measures.

### Procedure and Tasks

Informed consent was obtained from all participants before the initial screening and subjects were paid for their participation. Young adults were paid US $7 or $8 per hour (increased over the course of the study), whereas middle-aged and older adults were paid US $10 per hour. Data collection is described in detail in earlier papers from this project (Humes et al. 2009; Fogerty et al. 2010; Humes et al. 2010; Humes et al. 2013). In brief, a total of 40 psychophysical measures were obtained in hearing, vision, and touch with an emphasis on threshold sensitivity and temporal processing. The total testing time for each subject, including tests not reported in this article, was about 60 hr, typically divided into 40 sessions, each 90 min in duration. The project was approved by the Indiana University Bloomington Institutional Review Board.

#### Hearing Thresholds and Gap Detection Tests

For the auditory measures examined here, hearing thresholds in dB SPL and gap detection thresholds in milliseconds were obtained from three blocks of trials using a two-interval, two-alternative forced-choice measurement paradigm. Each block made use of interleaved adaptive tracking designed to concurrently estimate the 70.7 and 79.3 percent-correct points on the psychometric function (Levitt 1971). Both performance estimates (70.7 and 79.3%) were averaged across the three trial blocks such that each “threshold” was based on six performance estimates or 200 to 250 trials per threshold. Hearing thresholds were obtained for 500-msec pure tones at frequencies of 500, 1400, and 4000 Hz. Gap detection thresholds were obtained for 400-msec 1000-Hz bands of noise centered at either 1000 or 3500 Hz and presented at a level of 91 dB SPL. The 1000-Hz wide noise bands were presented in a complementary spectrally notched noise whose level was 12 to 15 dB lower than the noise band with the temporal gap. This noise served to mask any spectral cues from the introduction of the temporal gap.

#### Temporal Order Test

Four vowel stimuli, each of 70 msec duration, using recorded spoken voice of the form /p/-vowel-/t/ (pat, pet, pot, pit), were used to form two-item or four-item stimulus sequences (e.g., pat-pet, pat-pot-pit-pet; Humes et al. 2013). The vowels were low-pass filtered at 1800 Hz and presented at 83 dB SPL to minimize the influence of high-frequency hearing loss that was expected to be present in many of the older participants. Each vowel was identified in isolation by each subject with at least 90% accuracy. The method of constant stimuli was used to establish the 50%-correct point on the psychometric function relating the temporal separation of each vowel in the sequence (stimulus onset asynchrony, SOA, in milliseconds). An initial wide-range psychometric function was established using six widely spaced SOA values. This served to identify the likely threshold which was then explored further for three additional blocks of trials using smaller step sizes around the estimated threshold. The wide-range and the three narrow-range threshold estimates each made use of six SOA values, 10 trials per SOA, with randomization of SOA from trial to trial. The initial wide-range estimate was discarded and the final threshold was established by fitting a psychometric function to the data from the 180 trials for the narrow-range test conditions. All auditory testings were completed in a sound-attenuating test chamber and all stimuli were delivered via calibrated Etymotic Research 3A insert earphones.

#### Cognitive Tests

In addition, the full 13-scale Wechsler Adult Intelligence Scale-III, plus two optional incidental learning tests, were completed. An assistive listening device was available for use by the examiner when presenting the oral instructions or oral stimuli to any participants with impaired hearing. All cognitive testing were completed according to the manual (Wechsler 1995) individually in a quiet room.

The Wechsler Adult Intelligence Scale-III manual (Wechsler 1995) divides the subtests into four indexes; verbal comprehension, perceptual reasoning, working memory, and processing speed plus the optional incidental learning. The idea in the present article was to use cognitive concepts from memory theory and previously used concepts in studies of the relationship between auditory function and cognition (Rönnberg et al. 2011). In line with that, all subtests were analyzed to see if they could fit those types of categories. Working memory and processing speed were kept as they were, whereas the perceptual reasoning was excluded because no good match could be defined. Verbal comprehension has four subtests: vocabulary, similarities, information, and comprehension. These subtests all relate to using language from the knowledge component of semantic long-term memory (compare with Rönnlund et al. 2005). The Similarities subtest was excluded from the analysis because there were problems to find a good model, for several reasons that are elaborated on in the discussion. A vocabulary test is what is typically used to assess the knowledge component of semantic long-term memory. Therefore, these three subtests were used as indicators of semantic long-term memory. The paired-associate learning subtests include free recall which is typically used to assess the recall component of episodic long-term memory (compare with Nilsson et al. 1997; Rönnlund et al. 2003). Both paired-associate learning subtests have also been shown to be associated with other tests of episodic long-term memory (Wilson et al. 1982). Therefore, both these subtests were used as indicators of episodic long-term memory. An overview of all Wechler Adult Intelligence Scale-III subtests, which indices they belong to and which concepts they represent in the present article can be found in Table 1.

**TABLE 1.**
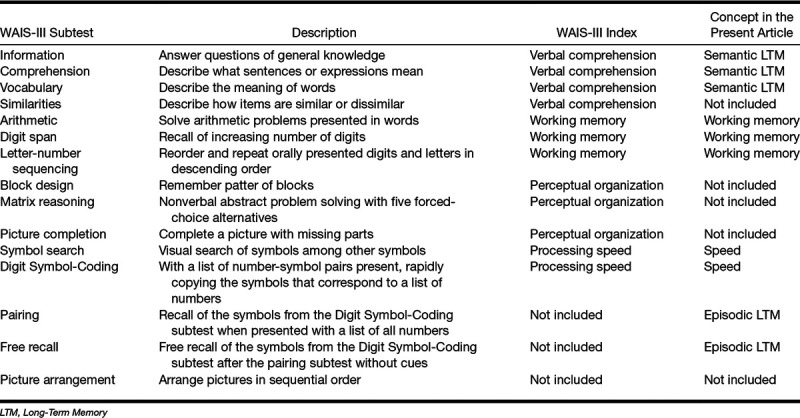
Wechsler Adult Intelligence Scale (WAIS-III) subtests and the concepts that they represent in the present article

### Statistical Testing Strategy

SEM was performed with AMOS 23.0 using maximum likelihood estimation. Factor scaling was achieved by setting one item per latent variable to a value of 1 in the pattern matrix. Figure 1 illustrates the basic model that was the starting point for the model optimization. The model consisted of age, the three auditory latent variables, and the four cognitive latent variables, where the latent variables had the measured indicators described earlier in the Materials and Methods. The optimization of the model used the modification index function in AMOS that suggests changes to the model based on statistical properties. These must be evaluated to make sure that they make sense from a theoretical point of view before being added to the model. Therefore, the optimization followed the rules described later, in order. As soon as a change had been made to the model, the optimization started again with the first rule and this was continued until no more changes could be made following the rules. The rules were as follows: 1. remove nonsignificant paths, starting with the one with highest *p*-value; 2. add paths between the latent variables and age, if suggested by modification index; 3. if suggested by the modification index, add paths from age to the measured indicators to adjust for differential item functioning (that the subtests have different associations with age) as recommended by the multiple indicators multiple causes approach (Muthén 1988); 4. add covariance between the measured auditory indicators or between the measured cognitive indicators (but not between one auditory indicator and one cognitive indicator). This is justified because these indicators are measured in a similar way and could reflect shared measurement variance. No covariance between the latent variables was allowed in the model.

There are no agreed upon set of fit indices when reporting SEM results, but there appears to be a relative consensus for using a variety of indices. Following a combination of recommendations (Hu & Bentler 1999; Schreiber et al. 2006), the fit of the model was evaluated using the following measures: The χ^2^, χ^2^/degrees of freedom (df), the root-mean-square error of approximation (RMSEA), the non-normed fit index (NNFI, also called Tucker Lewis Index, TLI), standardized root-mean-square residual (SRMR), and the comparative fit index (CFI). For χ^2^, *p* > 0.05 was used as the criterion, because the χ^2^ statistic is sensitive to sample size χ^2^/df < 2 (Tabachnick & Fidell 2007) was also used. There are different suggested cutoff values for RMSEA but following (Hooper et al. 2008) we chose the RMSEA < 0.07 criterion (Steiger 2007). CFI and NNFI values > 0.95 (Hu & Bentler 1999) were used as criteria for a good fit. For SRMR, the < 0.05 criterion (Byrne 1998) is chosen even if other researchers (Hu & Bentler 1999) have found a more lax criterion to be acceptable. Besides meeting all the above criteria, all path coefficients in the model also must be significant (*p* < 0.05) for the model to be accepted.

## RESULTS

Means, standard deviations, and correlations for all variables used in the modeling can be found in Table 2. The measured variables that are linked to the latent variables in the model show significant internal correlations (nearly all *r*s > 0.4), which suggest that the modeling will give reliable latent variables. It should be noted that there were also relatively high correlations between some of the variables not belonging to the same latent variable. This indicates that the cognitive constructs have some overlap. This is commented on in the Discussion.

**TABLE 2.**
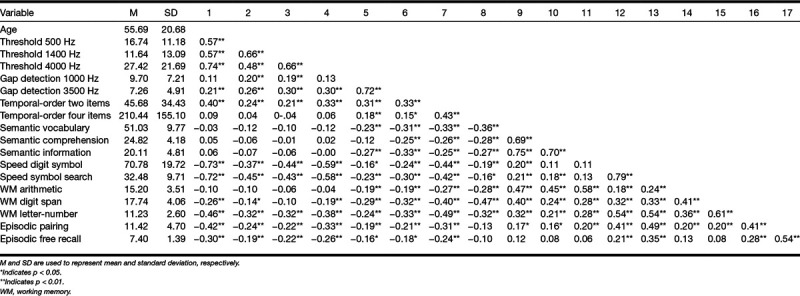
Means, standard deviations, and correlations for age and all auditory function and cognition variables used in the modeling

**Fig. 2.**
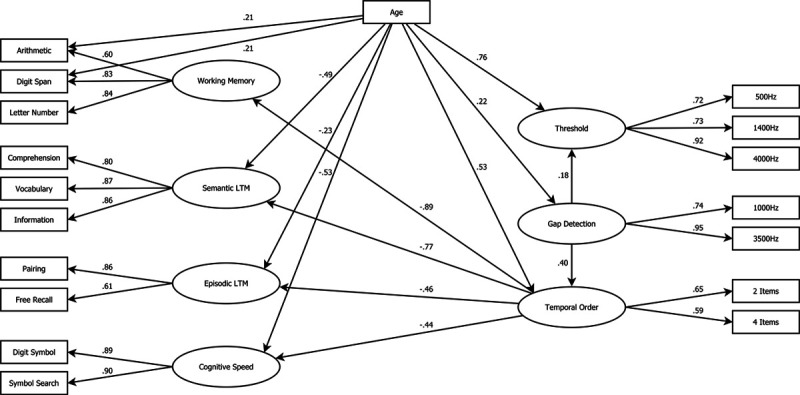
The final model of the relationships between age, the four auditory functions, and the three cognitive functions. Error terms and covariances are not shown in the figure.

The fit of the final model was good on all measures, χ^2^ (110) = 111.4, *p* = 0.44, χ^2^/df = 1.03, RMSEA = 0.008, NNFI = 0.999, CFI = 0.999, SRMR = 0.34. The final model can be seen in Figure 2, the direct and total effects are presented in Table 3, and the detailed description of all components of the model is available in Table R1 in Supplementary Digital Content, http://links.lww.com/EANDH/A508.

**TABLE 3.**
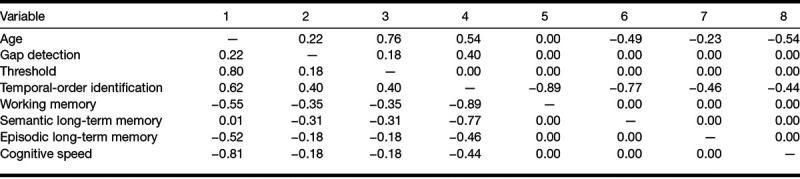
Standardized direct effects (upper right corner) and standardized total effects for age and the latent variables in the model. Effects not shown in the table (e.g., the effect of cognitive speed on age) are zero for both direct and total effects

The structure of the final model was different from the starting model. Gap detection affects both temporal-order identification and threshold. This can be understood as that gap detection is a more basic auditory function that also influences the other two abilities. There were also missing paths between some of the latent variables, notably gap detection and threshold had no direct effects on any type of cognitive function. The more detailed description of the final model that follows will start with the effect of age and then continue to the effect of auditory functions on cognition. In the description of the effects, Cohen’s rule of thumb for interpretation of effect sizes (Cohen 1988) will be used (0.10 to 0.30 small, 0.30 to 0.50 medium, and > 0.50 large).

Age has the largest total effects on threshold and cognitive speed (*β* about 0.80), large effects on temporal-order identification, working memory, and episodic long-term memory (0.51 < *β* < 0.63), small effect on gap detection (*β* = 0.22), and finally a nonsignificant effect on semantic long-term memory (*β* = 0.01). For most of the latent variables, the total effect of age was the sum of a large direct effect and a very small indirect effect. However, for working memory and semantic long-term memory, the pattern was different. For working memory, there were no direct effects of age, instead it was all due to the indirect effect. For semantic long-term memory, the direct and indirect effects went in different directions and canceled each other out in the total effect that was close to zero.

Gap detection had medium total effects on temporal-order identification, working memory, and semantic long-term memory (0.30 < *β* < 0.40) and small total effects on threshold, episodic long-term memory, cognitive speed, and semantic long-term memory ( *β* = 0.18). The total effect of gap detection was equal to the direct effect for temporal order and threshold, whereas for the cognitive variables, the direct effect was zero and all the total effects were due to the indirect effect via temporal-order identification. Temporal-order identification had no indirect effect on any other latent variable, but very large total and direct effects on working memory and semantic long-term memory ( *β* > 0.77), medium effects on episodic long-term memory and cognitive speed (0.44 < *β* < 0.46) but no effects on the other latent variables. Threshold had no effect on any of the cognitive latent variables in the model. To get an idea of the limitations of the final model, the modification index was inspected. It showed that much of the unexplained variance was related to how the indicators and errors of semantic long-term memory, working memory and gap detection were related to each other (e.g., a suggestion of adding a path from gap detection to arithmetic working memory, which does not make sense in relation to our theoretical understanding). This indicates that the theoretical construct has some overlap, which will be discussed later.

## DISCUSSION

The discussion will be based on the SEM model from the Results. That model is the best model given the data and the assumptions stated in the Introduction. We argue that our assumptions are plausible, but other assumptions can of course give other models. When different effects in the model are discussed, it is implied that it is dependent on the other parts of the model. We begin with a discussion of the auditory function effects on cognitive functions and later discuss the aging effects. Almost all paths in the models are a part of the same overall pattern: older age leads to lower auditory function and lower cognition, and lower auditory function leads to lower cognition. To avoid confusion about signs in the discussion, the magnitude (or absolute value) of the path coefficients will be used and the signs will be ignored.

Previous studies have consistently found correlations between auditory threshold and cognition (Lin et al. 2011; Rönnberg et al. 2011; Deal et al. 2015) in the range from *r* = 0.04 to 0.36, depending on the type of adjustments and methodology (Harrison Bush et al. 2015). In the present study, threshold had no significant direct or total effect on any of the cognitive functions. This was not in line with our prediction that thresholds will have an effect on episodic long-term memory, and semantic long-term memory, but not with working memory and cognitive speed. The smallest but still significant effect in the model was *β* < 0.18, which gives an indication of the magnitude of effects that can be detected in the present study. Another way to estimate this is to add the paths from threshold to the cognitive latent variables and keep them even if they are nonsignificant. Then, the magnitudes of the effects were 0.02 < *β* < 0.12. This indicates that, in the present study, the effect of threshold on various types of cognition (accounted for age and all other variables in the model) is closer to the lower end of what has been found in previous studies. This suggests that the effect of hearing on cognition is weaker in participants with normal hearing, which may be explained by a reduced variance in the threshold variable. Participants in the present study had relatively good hearing compared with previous studies. This weak association between threshold and cognition is, however, in line with findings that cognitive decline is dose-dependent on degree of hearing loss (Deal et al. 2015). Dose-dependency is also in line with many of the proposed hypotheses that suggest that sensory decline causes cognitive decline, such as the disuse hypothesis that the ease of language understanding model is based on (Rönnberg et al. 2013) and the information-degradation hypothesis (Schneider & Pichora-Fuller 2000; Pichora-Fuller 2003). The effects of confounding variables and measurement errors will be of similar magnitude independent of degree of hearing loss, which means that the effect of hearing on cognition will be harder to find for small variation of hearing function.

Gap detection had effects on both threshold and temporal-order identification, which indicates that it is a lower-order ability which is also important in the other types of tasks. An association between gap detection threshold and hearing threshold could be mediated by an ability to detect changes in signal amplitude. In gap detection, one is detecting a decrement in amplitude, the gap, whereas in hearing threshold one is detecting an increment in signal amplitude relative to an internal noise floor. This effect, however, is relatively weak (0.18). The effect of gap detection threshold on temporal-order identification is more straightforward and stronger (0.40). In both tasks, the minimum time interval needed between stimuli in a sequence of two (or more) sounds, noises for gap detection and vowels for temporal order, is determined. The tasks differ, however, simply to detect a gap for gap detection but to identify the vowels for temporal order. The spread of masking temporally to fill the gap or blur the sequence has been suggested as a common mechanism for at least the two-item temporal-order tasks, but the four-item task clearly involves additional higher level processes, including memory (Fogerty et al. 2016).

Gap detection and temporal-order identification both had the largest effects on semantic long-term memory and working memory, whereas the effects on episodic long-term memory and cognitive speed were smaller. Our predictions were that the associations for gap detection and temporal-order identification to the cognitive functions would follow the same predicted pattern as for thresholds: associations to episodic long-term memory, and semantic long-term memory, but no significant association with working memory and cognitive speed. This was based on the idea that deficits in auditory temporal-order identification and gap detection simply represent additional forms of degradation of the auditory input, akin to hearing thresholds. Because the predictions did not hold, and gap detection was modeled as a lower-order function, this affected the thresholds and temporal-order identification, which indicates a more complex relationship between the different auditory functions.

The stronger effects on cognition found for auditory functions other than threshold are consistent with the few previous studies investigating this. Cognition had stronger relationships with auditory temporal fine structure compared with hearing acuity in a study on hearing aid users (Rönnberg et al. 2016). Another study (Humes et al. 2013) used combined measures for different senses (hearing, vision, and touch) and found that temporal order for senses was more strongly related to cognition than sensory thresholds.

It could be argued that the stronger effect of auditory temporal-order identification on cognition is an artifact from that the task in itself demands a cognitive decision regarding identification of the order of the stimuli. In addition, this task demands a working memory component, where the stimulus must be remembered to allow for a correct answer, especially for the four-syllable sequences. Perhaps, it is no surprise that the strongest link to cognition was the one with working memory. There are of course no clean tasks that only measure one cognitive process or system, and even simple tasks such as the gap detection and temporal-order identification tasks demand cognitive decisions and memory involvement. These latter types of decisions do, however, reflect low levels of deliberate cognitive involvement (i.e., they are not taxing working memory or attention span) compared with the cognitive tasks used in the present study.

The aging effects on cognition in the present study are relatively consistent with previous findings. Age had the largest effect on cognitive speed, which starts to decline early and declines at higher rate than memory (Salthouse 2009). Semantic long-term memory is generally regarded as stable over the adult life span and peaking at a higher age than episodic long-term memory, even though there are differences between studies (see Rönnlund et al. 2005 for a review). This is consistent with the findings in the present study where the total aging effects on semantic long-term memory were close to zero. However, the indirect and direct effects were large which emphasizes that the level of cognitive abilities may be underestimated if hearing functions are not considered (compare with sensory effects on the cognitive test battery Montreal Cognitive Assessment, Dupuis et al. 2015). The degree of age-related decline in short-term memory and working memory is dependent on the type of task (Nilsson 2003) and is higher with higher cognitive demands (Salthouse 1994). It is also suggested that it could be mediated via cognitive speed (Salthouse 1994). In the present study, the age effect on working memory was large but not mediated via cognitive speed.

Aging had negative effects on each of the auditory functions included in the analyses: threshold, gap detection, and temporal-order performance. Associations between aging and hearing loss have been known for several decades and are so firmly established as to be described in an International Standards Organization standard (ISO 2017). Humes and colleagues (2012) provided a comprehensive review of the literature on the effects of aging on a wide range of auditory functions and noted that the observed age effects on gap detection and temporal-order performance could not be explained simply as secondary effects of hearing loss. Whereas 15 such studies had been conducted for gap detection in the period from 1988 to 2011, only 5 studies examined temporal-order processing over this same period. Nonetheless, for both gap detection and temporal-order processing, age effects have been clearly demonstrated. Our results replicate these aging effects.

The links observed between auditory performance and cognitive function have a variety of practical implications. For example, those older adults with auditory deficits, hearing loss, or temporary-processing deficits may be more likely to have deficits in cognitive function. Although cause-and-effect cannot be inferred from the observed associations, these associations should be corroborated by other studies, especially longitudinal studies, and then there may be implications for intervention. Intervention to alleviate auditory deficits may improve cognitive function immediately and, perhaps, could forestall subsequent cognitive decline over a longer period of time. Further research is needed, however, before such implications can be established.

There are limitations with this study. One limitation, which is discussed in the Introduction, is that SEM statistically tests a causal model but this must be backed up by theoretical reasoning. Given the literature review in the Introduction, the proposed causal model is reasonable, but longitudinal studies are needed to further validate this. Another limitation is that other variables that were not measured in this study could affect the relationships between the latent variables. Candidates for such variables could be with different health issues that has been linked to hearing loss, for example, diabetes (Mitchell et al. 2009), cardiovascular disease (Lin et al. 2008), hypertension (Kiely et al. 2012), and depression (Huang & Rong 2010). Diabetes has also been found to have a negative effect on auditory temporal-order identification (Humes 2016). It has also been suggested that social psychological factors may modulate auditory and cognitive functioning (Pichora-Fuller 2016). It is reasonable to think that some of the unmeasured variables in the present study influence the estimated relationships between auditory and cognitive functions to some extent. However, it is still very probable that the general conclusion of the present article, that there are different effects of auditory function on cognition (and/or vice versa) depending on the type of auditory function and cognition, is valid.

As is the case in a theoretical reasoning, the constructs used in this study are discussed as separate entities. In reality, they are partly correlated, as can be seen in Table 1. This is the case for cognitive constructs in general and for working memory in particular. This is not surprising as almost all cognitive tasks have some type of working memory demand, for example, remembering the instructions, and the lack of clean tasks that taps into only one construct. The unexplained variance in the model was mainly found as different suggested associations between working memory, semantic long-term memory, and gap detection. This also indicates that especially the cognitive constructs could be constructed in other ways. With this caveat in mind, we nonetheless think that the division used in the present article still supports the general idea that the association between age, auditory function, and cognition looks different depending on the type of variable used to represent auditory function and cognition.

Finally, it is important to note that the collection of the auditory data by Humes et al. (2013), used in this modeling, took great care to minimize the influence of hearing loss on other auditory and cognitive measures. For auditory gap detection, for example, the frequencies and levels of the stimuli were chosen to be within the audible region of the participants. Likewise, the vowel stimuli used for the temporal-order identification tasks were low-pass filtered and presented at relatively high presentation levels to once again minimize the influence of high-frequency hearing loss. Further, for the temporal-order tasks, participants had to identify each vowel token in isolation with at least 80% accuracy before they were placed into sequences. Even for the cognitive assessments, those participants presenting with hearing difficulty were instructed and tested with the aid of an assistive listening device as needed to minimize the direct influence of inaudibility on the cognitive measures. To the extent that other studies do not control for the loss of audibility associated with the presence of high-frequency hearing loss in many older adults, then the model which emerges in those studies showing associations among those measures may differ. It is not hard to imagine scenarios in which the performance on auditory tests other than threshold may be totaling determined by the (in)audibility of the auditory stimuli used in those tasks. This notion is easily extended to cognitive measures, such as the Wechsler Adlut Intelligence Scale-III, which may make liberal use of oral instructions and auditory (spoken) stimuli.

## CONCLUSIONS

The novel aspect of this study was to use nonthreshold auditory tasks to model how age and auditory functions affect cognition. One model with the measured variable age, three latent auditory variables (threshold, gap detection, and temporal-order identification) that affected four latent cognitive variables (episodic long-term memory, semantic long-term memory, speed of processing, and working memory) was evaluated and found to have a good fit to the data. The overall take-home message is that the effects of age and auditory function on cognition looks different depending on the type of variable used to represent auditory function and cognition. This is an important finding that emphasizes the need to include different types of auditory functions, other than hearing thresholds, as has been the case traditionally, when investigating the associations with cognition.

The results found a model where the effects on the latent cognitive variables were largest for temporal-order identification, followed by gap detection, but were nonsignificant for threshold once the direct effect of age had been allowed for. The auditory effects on the latent cognitive measures were largest for semantic long-term memory and working memory but weaker for episodic long-term memory and cognitive processing speed. Age has the largest total effects on threshold and cognitive speed, large effects on temporal-order identification, working memory, and episodic long-term memory, small effect on gap detection, and nonsignificant, and close to zero effect on semantic long-term memory.

## ACKNOWLEDGMENTS

This study was financed by an excellence grant [349-2007-8654] from the Swedish Research council; a grant [2012-1693] from The Swedish Research Council for Health, Working Life and Welfare (to J.R.) and a grant [R01 AG008293] from the National Institute on Aging (to L.H.). The authors have no conflicts of interest to disclose.

## Supplementary Material
