# Supplementary material for: Different Associations between Auditory Function and Cognition Depending on Type of Auditory Function and Type of Cognition
Source: Ear Hear. 2019 Aug 23;40(5):1210–9. doi: 10.1097/AUD.0000000000000700 (PMC6706331; doi:10.1097/AUD.0000000000000700)
Supplement: Supplementary file 1 [file aud-40-1210-s001.pdf]

Online Supplement Table

Unstandardized coefficients, standardized coefficients, and p-values for paths, covariances and variances for the final model.

Part of model			Unstandardized coefficient	Standardized coefficient	*p*-value
Paths					
Gap Detection	<---	Age	0.06	0.22	**
Temporal Order	<---	Age	0.58	0.54	***
Threshold	<---	Age	0.30	0.76	***
Episodic	<---	Age	-0.05	-0.23	*
Semantic	<---	Age	0.08	0.49	***
Speed	<---	Age	-0.23	-0.54	***
Arithmetic	<---	Age	0.04	0.21	**
Digit Span	<---	Age	0.04	0.21	**
Temporal Order	<---	Gap Detection	1.67	0.40	***
Threshold	<---	Gap Detection	0.28	0.18	***
Episodic	<---	Temporal Order	-0.09	-0.46	***
Semantic	<---	Temporal Order	-0.12	-0.77	***
Speed	<---	Temporal Order	-0.17	-0.44	***
Working Memory	<---	Temporal Order	-0.09	-0.89	***
Gap Detection 1000Hz	<---	Gap Detection	1.00	0.74	-
Gap Detection 3500Hz	<---	Gap Detection	0.90	0.95	***
Temporal Order 2 items	<---	Temporal Order	1.00	0.65	-
Temporal Order 4 items	<---	Temporal Order	0.93	0.59	***
Threshold 500Hz	<---	Threshold	1.00	0.72	-
Threshold 1400Hz	<---	Threshold	1.18	0.73	***
Threshold 4000Hz	<---	Threshold	2.48	0.92	***
Free Recall	<---	Episodic	0.21	0.61	***
Pairing	<---	Episodic	1.00	0.86	-

Comprehension	<---	Semantic	1.00	0.80	-
Information	<---	Semantic	1.24	0.87	***
Vocabulary	<---	Semantic	2.56	0.87	***
Digit Symbol	<---	Speed	2.03	0.89	***
Symbol Search	<---	Speed	1.00	0.90	
Arithmetic	<---	Working Memory	0.96	0.60	***
Digit Span	<---	Working Memory	1.52	0.83	***
Letter Number	<---	Working Memory	1.00	0.84	-
Covariances					
Error Temporal Order 2 items	<-->	Error Temporal Order 4 items	263.49	0.36	***
Error Threshold 500Hz	<-->	Error Threshold 1400Hz	19.97	0.29	*
Error Threshold 500Hz	<-->	Error Threshold 4000Hz	-44.68	-0.67	***
Error Comprehension	<-->	Error Arithmetic	3.68	0.48	***
Error Comprehension	<-->	Error Symbol Search	2.10	0.19	*
Error Information	<-->	Error Arithmetic	6.14	0.85	***
Error Information	<-->	Error Pairing	1.61	0.27	*
Error Vocabulary	<-->	Error Digit Span	3.57	0.27	**
Error Vocabulary	<-->	Error Arithmetic	8.11	0.57	***
Error Digit Span	<-->	Error Free Recall	-0.63	-0.21	**
Error Symbol Search	<-->	Error Pairing	3.14	0.30	*
Error Digit Symbol	<-->	Error Free Recall	-3.00	-0.30	***
Variances					
Age			424.58		***
Error Temporal Order 2 items			677.21		***
Error Temporal Order 4 items			784.96		***
Error Gap Detection 1000Hz			23.15		***
Error Gap Detection 3500Hz			2.56		0.31
Error Threshold 500Hz			58.82		***
Error Threshold 1400Hz			80.37		***

Error Threshold 4000Hz	74.84	**
Error Comprehension	6.44	***
Error Information	5.79	***
Error Vocabulary	22.56	***
Error Arithmetic	9.02	***
Error Digit Span	7.54	***
Error Letter Number	2.00	***
Error Symbol Search	18.72	***
Error Digit Symbol	82.26	***
Error Pairing	6.07	*
Error Free Recall	1.21	***
Error Temporal Order	227.39	***
Error Gap Detection	26.64	***
Error Threshold	21.37	***
Error Episodic	10.17	***
Error Semantic	7.10	***
Error Speed	17.30	***
Error Working Memory	1.03	**

Note. For *p*-values, $p < .05$ is marked with *, $p < .01$ is marked with **, $p < .001$ is marked with ***, and where *p* -values were not available is marked with -.
